# Numerical Investigation on Thermal Conductivity of Graphene Foam Composite for Thermal Management Applications

**DOI:** 10.3390/ma17133300

**Published:** 2024-07-04

**Authors:** Rongyao Zhou, Yuexia Lv, Tingting Du, Jinpeng Bi

**Affiliations:** 1 School of Mechanical Engineering, Qilu University of Technology (Shandong Academy of Sciences), Jinan 250353, China; 2 Shandong Institute of Mechanical Design and Research, Jinan 250031, China; 3 School of Energy and Power Engineering, Shandong University, Jinan 250061, China; 4 Shenzhen Research Institute of Shandong University, Shenzhen 518057, China

**Keywords:** graphene foam composite, thermal conductivity, finite element method, CVD

## Abstract

Graphene foam prepared by the chemical vapor deposition method is a promising thermal interfacial material. However, the thermal properties of graphene foam highly depend on the experimental fabrication conditions during the chemical vapor deposition process. Aiming to reveal how to prepare the appropriate graphene foam for the various thermal management scenarios, the influence of experimental conditions on thermal properties of graphene foam was investigated. Furthermore, the contribution of thermal conductivity and thermal radiation to the effective thermal coefficient of graphene foam was carried out for comparison. The research results showed that the porosity and the cross-section shape of the struts of the growth template were two critical factors affecting the thermal transport of graphene foam, especially with the increase of temperature. In addition, the deposition time of graphene determined the wall thickness and affected the thermal conductivity directly. The thermal radiation contributed more than thermal conductivity when the temperature climbed continuously. Comparatively, the effective thermal coefficient of graphene foam composite with high porosity and circular-shape struts was much superior to that of others at high temperature. The research findings provide important guidance for graphene foam fabrication and its applications in the field of thermal management.

## 1. Introduction

With the rapid development of increasingly compact and multi-functional electronics, excessive heat flux is inevitably becoming the most urgent issue, which leads to performance degradation, component failure, and even safety hazards. Therefore, effective thermal management and heat dissipation play a crucial role in addressing the above problems. Correspondingly, a variety of innovative thermal solutions has been developed to enhance the thermal performance of electronics, including advanced heat sinks, thermal interface materials, cooling systems, and high thermal conductive phase-change materials [1,2,3,4]. Graphene, a typical two-dimensional (2D) crystal with a layer of carbon atoms packed into a honeycomb lattice, has attracted most attentions in recent years due to its unique characteristics of superior electrical and thermal conductivities, high mechanical rigidity, and controllable functionalization [5]. Therefore, it has been extensively applied in various scenarios, especially in energy storage and conversion systems, including electrochemical sensors [6,7], composite materials [8,9], energy storage devices [10,11], adsorbents [12,13], and drug delivery [14,15]. However, the fabrication of extremely thin graphene with one atomic layer is difficult to realize for mass production and the applications are limited. In addition, the strong π-π interactions and van der Waals forces between adjacent graphene sheets result in agglomeration or accumulation phenomena, which significantly reduce the specific surface area and seriously damages corresponding performance [16].

One of the most effective solutions is to develop three-dimensional (3D) graphene-based materials with porous structures, which not only retains the intrinsic properties of graphene, but also acquires the advantages of high porosity, large specific surface area, and low density of porous materials [17]. Graphene foam (GF) is a highly porous and light material with 3D interconnected graphene networks that is typically synthesized via the chemical vapor deposition (CVD) method. Attributed to its excellent mechanical strength, flexibility, and high conductivity [18], GF is a good candidate substrate for energy storage and conversion applications, working as an excellent thermal conductive material [19,20], effective thermal insulation material [21], and thermal interface material [22,23]. The structure and performance of GF highly depends on the fabrication parameters of the CVD method, including the type of growing substrates [24,25], growing temperature [26], carbon source concentration [27], and etching solution [28].

Effective thermal conductivity is one of the most important parameters to evaluate the thermal properties of graphene foam, which is contributed by solid thermal conduction and radiation, as well as the thermal conduction and convection of the medium filling the void spaces. Pettes et al. [28] experimentally measured the thermal conductivity of GF, which was increased from 0.26 to 1.70 W·m^−1^·K^−1^ by using different etchants. Qi et al. [29] fabricated a novel 3D hierarchical GF macrostructure by filling the pores of GF with hollow grapheme networks. Their experimental results showed that the thermal conductivity of the hierarchical macrostructures was increased to 2.28 W·m^−1^·K^−1^, which was 744% higher than that of the pure paraffin wax-based composite phase-change materials. Gao et al. [30] experimentally studied the thermal properties of a free-standing GF skeleton and found that the thermal conductivity was within the range of 520–550 W·m^−1^·K^−1^, which could be attributed to the effective heat dissipation network of the interconnected skeleton. The thermal properties of free-standing GF highly depended on the temperature, and its thermal conductivity was increased from 0.3 W·m^−1^·K^−1^ to 1.5 W·m^−1^·K^−1^ when the temperature varied from 310 K to 440 K [31]. As mentioned above, the thermal conductivity is valued differently depending on different experiments. In addition, the experimental measurement did not uncover the reason for the various experimental thermal conductivity and the contribution of different heat transfer methods.

A variety of numerical simulation models has been proposed to predict the thermal properties of GF, mainly including molecular dynamics simulation and the finite element method. For porous foam materials, molecular dynamics simulations mainly provide insights into thermal motions and thermal conduction pathways at a molecular level, which are helpful to understand the micro-scale thermal conduction mechanisms of the materials. Khosravani et al. [32] applied molecular dynamics simulation to investigate the thermal conductivity of a polymer composite consisting of graphene foam/polydimethylsiloxane (GF/PDMS). It was found that the thermal conductivity of this composite was increased by 69% compared to pure PDMS. Chen et al. [33] systematically investigated the anisotropic thermal transport of carbon foams composed of six-winged graphene nanoribbons by using the equilibrium molecular dynamics simulation method. The results indicated that the significant anisotropic behavior could be attributed to the orientation-dependent group velocity of long-wavelength phonons. Furthermore, the interfacial thermal conductivity could be enhanced by increasing the ambient temperature or external pressure. Due to high computational costs and limited simulation scales, it is still difficult to apply this method to study the thermal properties of porous materials at a macroscopic scale, or investigate the radiative heat transfer mechanisms.

The simulations based on the finite element method consider the overall material structure, pore morphology, and heterogeneity. Therefore, it is possible to quantitatively analyze the thermal conductivity of porous foam materials, and describe the coupling mechanism between solid thermal conduction and radiative heat transfer. However, there is limited research/literature studying the numerical simulation of thermal conductivity of GF using the finite element method. Xiong et al. [34] validated the dynamic thermal behavior of compressed flexible GF based on the finite element method, finding that the in-plane thermal conductivity increased while the cross-plane thermal conductivity decreased with compression. Furthermore, a spring model was proposed to predict the dynamic thermal conductivity of GF under compression. Zhang et al. [35] developed a finite element model to predict the thermal behavior of a GF/PDMS polymer composite, and further investigated the influences of GF size, contact, and interfacial resistance on the thermal conductivity. Numerical results showed that the thermal conductivity of the polymer composite filled with graphene was increased by increasing radius and reducing length of GF struts. The representative volume element for the composite modeling is a typical Kelvin octahedron composed of quadrilaterals and hexagons. However, the pore structure of GF is predominantly composed of dodecahedral structures. Correspondingly, the present study established a physical model based on the dodecahedral structure of GF. The finite element method was used to investigate the influence of porosity and temperature on the effective thermal conductivity of GF under the conditions of solid thermal conduction, as well as conduction radiation-coupled heat transfer. This research may guide the preparation process of GF to obtain the required GF composite with potential thermal properties.

## 2. Methodology

### 2.1. Design of Physical Structure

During the fabrication process of GF via chemical vapor deposition, the morphology and structural parameters of the growth template directly determine the structure of GF, thereby affecting its thermal properties. Now, the most commonly used templates in experiments include nickel foam [29], copper foam [36], aluminum foam [25], and so on. Although all these metal foams own a continuous network, it is distinct in cross-section shape, struts diameter, and porosity due to the difference in surface property and preparation variables.

**Figure 1:**
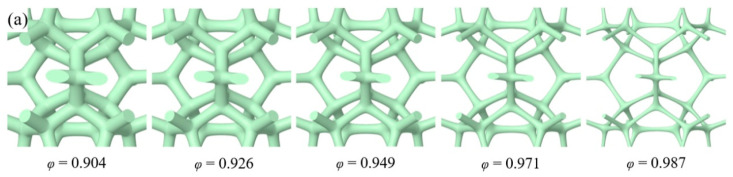

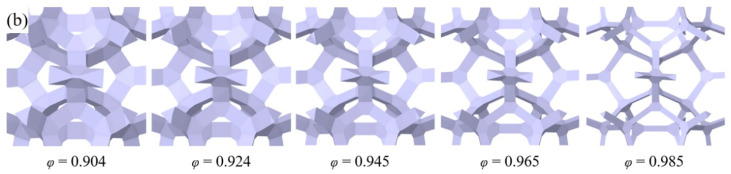
Physical models of GF of different porosities (**a**) with circular-shape cross section struts; (**b**) with triangular-shape cross section struts.

In order to investigate the influence of different templates on the thermal properties of GF, this research established physical models with triangular-shape and circular-shape struts and with different strut diameters, as shown in Figure 1. The topology skeletons were built by 3dMax 2021 and SpaceClaim 2021 R1 deriving from the real GF prepared by the CVD method. The state of the nickel foam substrate that had not been etched off during the preparation of graphene foam by CVD method was selected as the research object. The graphene was deposited on the surface of the nickel foam, showing an extremely thin layered structure. In order to reduce the modeling and computational burden, shell conduction was used to characterize the graphene units deposited on the surface of the nickel foam. The porosity of the GF composite ranged from 0.904–0.985 for triangular-shape struts and from 0.904 to 0.987 for circular-shape struts. The porosity is the ratio of the void volume to the total volume of the cell. As the physical structure remained the same, the different porosities were obtained by adjusting the equivalent diameter of the struts.

### 2.2. Heat Transfer of GF Composite

Generally, heat transfer of open-cell foams is via thermal conduction of the solid skeleton and the filling material in pores. If the filling material is gas, it also takes consideration of heat convection between the solid skeleton and the filling gas, the thermal radiation between the surfaces of struts, as well as between the skeleton and the gas. As the pore size of GF composite is normally less than 500 μm, the natural heat convection in the pores can be neglected. Meanwhile, the thermal radiation can also be ignored when the temperature is below 400 K [37]. It was reported that when the temperature was above 800 K, the effective heat transfer coefficient of metal foams was three times higher than that at a room temperature [38], which revealed the integral role of thermal radiation. Therefore, with the temperature rising, the heat transfer of GF composite should consider the co-contribution of thermal conduction and thermal radiation.

For the foam of graphene deposited on the Ni skeleton, the heat transfer at a room temperature is the thermal conduction of the shell-like graphene, the interior Ni solid, and the filled gas in the pores. As the deposited graphene contacts with Ni solid, the heat transfer consists of not only the thermal conduction of graphene and Ni separately, but also the thermal conduction between graphene and Ni. With consideration of the graphene structure and the heat transfer property, the method of thin-shell element was adopted here in the numerical simulation. Herein, the total heat transfer (*Q_t_*) contributed by the thermal conduction (*Q_c_*) can be expressed as:

(1) $$Q_{t}=Q_{c}$$

With the rise of temperature, thermal radiation should be taken into account. Since the emissivity and the absorption of gas are normally small, the thermal radiation only considers the radiation between solid surfaces. Therefore, the total heat transfer contributed by the thermal conduction and the thermal radiation (*Q_r_*) is expressed as:

(2) $$Q_{t}=Q_{c}+Q_{r}$$

### 2.3. Boundary Conditions and Governing Equations

**Figure 2:**
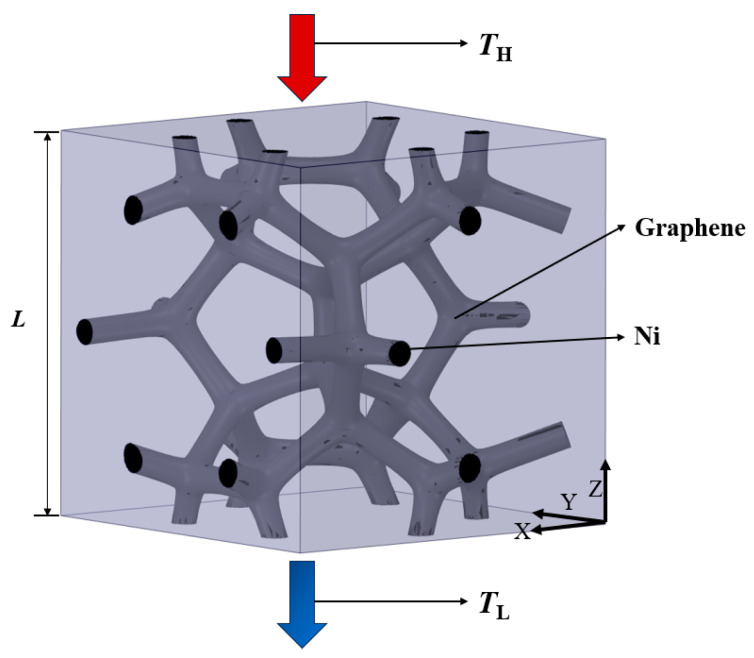
The computational domain and the boundary conditions.

The cells of GF composite fabricated by the CVD method are highly uniform. In this research, a cell was adopted to investigate the thermal properties of GF composite. The size of the unit including the characterized cell and the air domain was 1 mm × 1 mm × 1 mm, as shown in Figure 2. The heat flows from the top surface with the high temperature (*T_H_*) to the bottom surface with the low temperature (*T_L_*). The other surfaces are symmetric and thermal isolated without slip. The relevant assumptions are as follows:

(1) Because the CVD-based GF is mostly used as a thermal interface material, the heat transfer along the direction of thickness is much more important. As the thickness of the GF composite is much smaller, the heat flow of the GF composite is set as a one-dimensional transfer in the Z-axial direction;
(2) The thermal conduction of the solid skeleton is isotropic;
(3) The thermal conductivity of solids is constant.

The heat transfer in the GF composite satisfies the equation of conservation of energy. When the temperature is lower than 400 K, the temperature field inside the steady-state GF composite which has no internal heat source and only takes thermal conduction into consideration satisfies the following differential equation:

(3) $$\mathrm{div}\left(k\;\mathrm{grad}T\right)=0\;\left(T<400\;K\right)$$

where *k* is the thermal conductivity, W·m^−1^·K^−1^; *gradT* is the temperature gradient.

When the temperature is higher than 400 K, in which case the thermal radiation effect cannot be neglected, the energy equation of the GF composite for the steady-state combined heat transfer is expressed by the following equation:

(4) $$\mathrm{div}\left(k\;\mathrm{grad}T-q_{r}\right)=0\;\left(T\geq 400\;K\right)$$

where *q_r_* is the density of the radiation heat flow, W·m^−2^.

For a one-dimensional heat transfer system where the heat is transferred along the Z direction, the differential equation can be simplified as:

(5) $$\frac{\partial }{\partial z}\left(k_{z}\frac{\partial T}{\partial z}\right)=0$$

where *k_z_* is the thermal conductivity in the Z direction, W·m^−1^·K^−1^.

When the temperature is lower than 400 K, the heat transferred along the Z direction is composed only of solid thermal conduction and air thermal conduction:

(6) $$q_{z}=q_{c}$$

where *q_z_* is the heat flux in the Z direction, W·m^−2^; *q_c_* is the conduction heat flow, W·m^−2^.

When the temperature is higher than 400 K, the heat transferred along the Z direction is contributed by both thermal conduction and radiation, expressed as:

(7) $$q_{z}=q_{c}+q_{r}$$

where *q_r_* is the radiation heat flow, W·m^−2^.

According to Equations (5)–(7), the heat flux in the Z direction can be expressed by the temperature gradient as:

(8) $$q_{z}=q_{c}=-k_{ec}\frac{\partial T}{\partial z}\left(T<400\;K\right)$$

(9) $$q_{z}=q_{c}+q_{r}=-\left(k_{ec}+k_{er}\right)\frac{\partial T}{\partial z}\left(T\geq 400\;K\right)$$

where *k_ec_* is the conductive thermal conductivity, W·m^−1^·K^−1^; *k_er_* is the radiative thermal conductivity, W·m^−1^·K^−1^.

The temperature gradient is used as the thermal boundary conditions, expressed as follows:

(10) $$\begin{array}{c} T_{Z=0}=T_{H}\\ T_{Z=L}=T_{L} \end{array}$$

where *T_H_* and *T_L_* are the temperature at the bottom and top surfaces of the GF composite respectively, K.

Due to the complex pore structure of the GF composite, in order to improve the accuracy without increasing the computational workload, the surface-to-surface (S2S) radiation model was used for the calculation of radiative heat transfer. The S2S model takes into account radiative exchange on a gray diffuse surface. In the S2S model, any absorption, emission, or scattering of radiation by the medium can be ignored. The heat flux leaving a given surface *k* is composed of emitted and reflected energy as follows:

(11) $$q_{r}{=q}_{out,k}=\varepsilon_{k}\sigma T_{k}^{4}+\left(1-\alpha_{k}\right)q_{in,k}$$

The energy flux leaving the surface *k* via radiation is *q_out,k_*, W·m^−2^. The emissivity and absorptivity of surface *k* are *ε_k_* and *α_k_*, respectively. *T_k_* is the surface temperature and *q_in,k_* is the energy flux incident on the surface from other surfaces, W·m^−2^. The incident energy is determined by the position, the direction, and the relative angle of two surfaces. The surface-to-surface “view factor” *F_kj_* is introduced to illustrate this relationship. The incident energy flux can be expressed as:

(12) $$A_{k}q_{in,k}=\sum_{j=1}^{N}\;{A_{j}F}_{kj}q_{out,j}$$

where *A_k_* and *A_j_* are the area of surface *k* and *j*, m^2^; *q_out,j_* is the energy flux leaving the surface *j*, W·m^−2^. In detail, the view factor is expressed as:

(13) $$F_{kj}=\frac{1}{A_{j}}\int_{A_{k}}\;\int_{A_{j}}\;\frac{\cos\theta_{k}\cos\theta_{j}}{\pi r^{2}}\delta_{kj}dA_{k}dA_{j}$$

where *θ_k_* and *θ_j_* are the angle between the normal of each surface and the line of *dA_k_* and *dA_j_*; *r* is the distance between *dA_k_* and *dA_j_*, m; *θ_kj_* is determined by the visibility of *dA_j_* to *dA_k_*. If *dA_j_* to *dA_k_* is visible, *θ_kj_* can be 1, otherwise 0. Based on the reciprocity relationship of the view factor (*A_k_F_kj_* = *A_j_F_jk_*) and Kirchoff’s law (*ε_k_* = *α_k_*), it can be derived that:

(14) $$q_{out,k}=\varepsilon_{k}\sigma T_{k}^{4}+\left(1-\varepsilon_{k}\right)\sum_{k=1}^{N}\;F_{kj}q_{out,j}$$

The view factor is solved by the S2S radiation model embedded in Fluent, and the effective radiation of each surface can be obtained based on the angular coefficient, thus obtaining the radiative heat transfer of each surface. The total heat flux through the GF composite can be calculated by solving the view factor and the energy equation at the same time. Effective thermal conductivity *k_eff_* based on Fourier’s Law is expressed by:

(15) $$k_{eff}=\frac{q_{z}}{\frac{\left(T_{H}-T_{L}\right)}{L}}$$

where *k_eff_* is the effective thermal conductivity, W·m^−1^·K^−1^; *L* is the thickness of the GF composite, m.

Although the skeleton surface of the GF composite was not smooth because of the inhomogeneity of deposition in the experiments, the influence of the view factor on the radiative heat transfer was more significant than that of the surface roughness. Therefore, all surfaces were assumed to be smooth in the numerical simulation.

ANSYS Fluent 2021 R1 was used to solve the heat transfer process inside the GF composite. The steady-state pressure-based solver was used for calculation, and second-order upwind was used to discretize the thermal conduction equation inside the GF composite. The SIMPLE algorithm was adopted for the pressure velocity coupling and the PRESTO method was used to discretize the pressure equation. By solving the energy equations with and without considering radiation, the temperature distribution of the GF composite and the total heat flux through the GF composite were obtained, which could be further used for the calculation of effective thermal conductivity. Table 1 presents the thermophysical properties of materials used in the simulation.

### 2.4. Verification of Grid Independence

**Figure 3:**
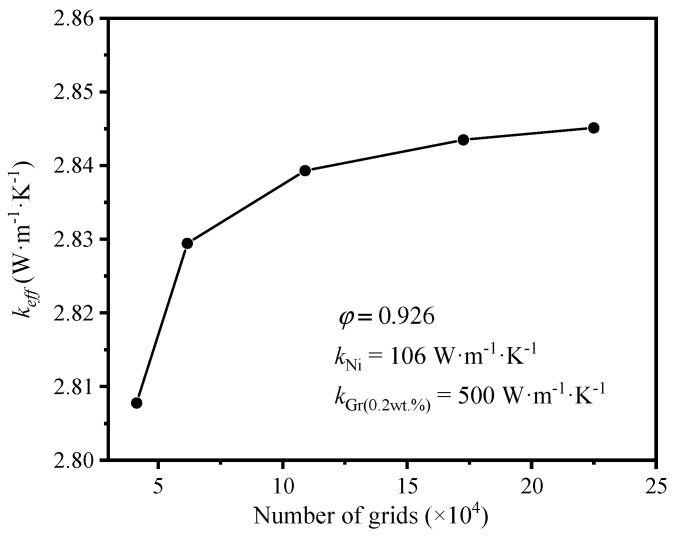
Variation of the effective thermal coefficient with the porosity.

Unstructured polyhedral mesh was adopted for grid generation due to the complexity of the model generated by Fluent meshing. The grid independence was verified by selecting the model with the circular-shape struts and a porosity of 0.926. The high-temperature surface was set as 320 K, and the low-temperature one was 300 K. The filled gas was air. The mass fraction of graphene was 0.2%. Five-set grids were conducted in the verification. It was found that with the increase of grid numbers, the effective thermal coefficient climbed firstly, and then plateaued, as shown in Figure 3. When the grid numbers were more than 1,090,007, the deviation of effective thermal coefficient was less than 0.3%, which was acceptable. In order to balance the accuracy and the consumed time of calculation, the third-set grid (number of grids = 1,090,007) was selected in this numerical simulation.

### 2.5. Numerical Method Validation

**Figure 4:**
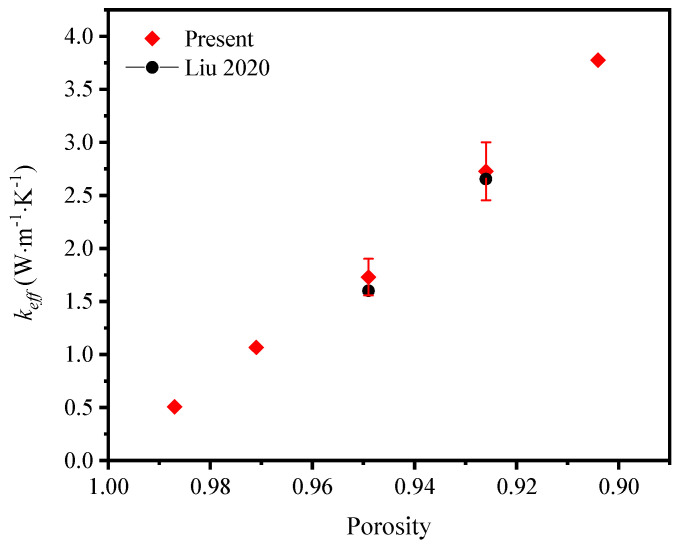
Comparison between the numerical and experimental results [39].

To validate the feasibility of the method, the numerical thermal conductivity of the graphene–nickel/epoxy composite was compared with the experimental results [39]. The temperature of the top and bottom surfaces and the composite materials of the skeleton was kept at the same temperature. Only the filling material was changed into epoxy resin. The experiment showed the effective thermal coefficient increased from 1.60 W·m^−1^·K^−1^ to 2.65 W·m^−1^·K^−1^ with the increase of graphene layers, which was determined by the deposition time. According to the XRD and Roman spectral intensity test on the graphene growth patterns at different reaction times, the mass fraction of graphene deposition was converted to the equivalent porosities of the physical models when the deposition rate remained the same. Then, it was calculated that the effective thermal coefficient of the GF composite rose the more graphene was deposited on the skeleton; namely, with the decrease of porosity, which agreed with the experimental conclusions. When the porosity decreased from 0.949 to 0.926, the effective thermal coefficient increased from 1.73 W·m^−1^·K^−1^ to 2.73 W·m^−1^·K^−1^ with an acceptable error (<5%) compared with the experimental results, as shown in Figure 4. The reason for a larger numerical result is that the heat process in the simulation was optimized and some of the boundary conditions were ideal. It can be concluded that the numerical method was a feasible one with which to investigate the thermal properties of GF composite.

## 3. Results and Discussion

### 3.1. The Thermal Properties and the Influencing Factors at Room Temperature

In the preparation of GF by the CVD method, the properties often vary with the different growth templates, porosities, and mass fractions of graphene. To learn the impact of variables in the experimental procedure on the effective thermal coefficient, the cross-section shape of struts, the porosity, and the deposition thickness of graphene were investigated.

#### 3.1.1. The Impact of the Growth Template on Thermal Properties

**Figure 5:**
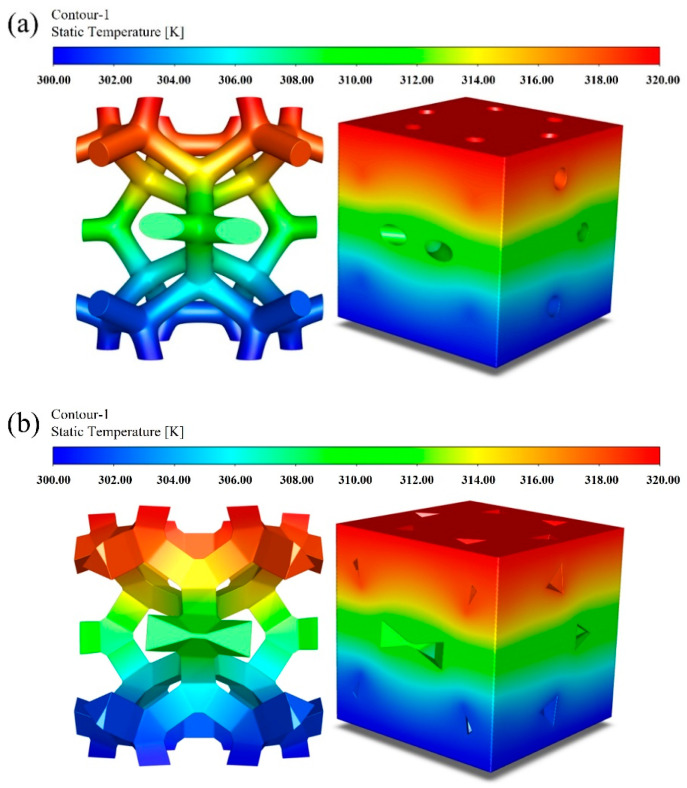
Temperature distribution of the solid and fluid domains in different cross-section shapes of struts: (**a**) circular-shape struts; (**b**) triangular-shape struts.

The growth template mainly differs with the porosity and the cross-section shape. At the same porosity (*φ* = 0.904), the temperature fields of GF composites with circular-shape and triangular-shape struts are shown in Figure 5. Both show the temperature gradient distribution along the longitude of the skeleton. There is an obvious temperature drop at the junction of struts. Correspondingly, the temperature distribution of the fluid domain seems the same as that of the solid domain since it is affected significantly by the foam skeleton, but the influence of the cross-section shape on the temperature distribution is remarkable, especially at the junction. The distribution is more uniform in the foam with circular-shape struts than that with triangular-shape struts, which demonstrates the impact of the cross-section shape on the thermal property to some degree.

**Figure 6:**
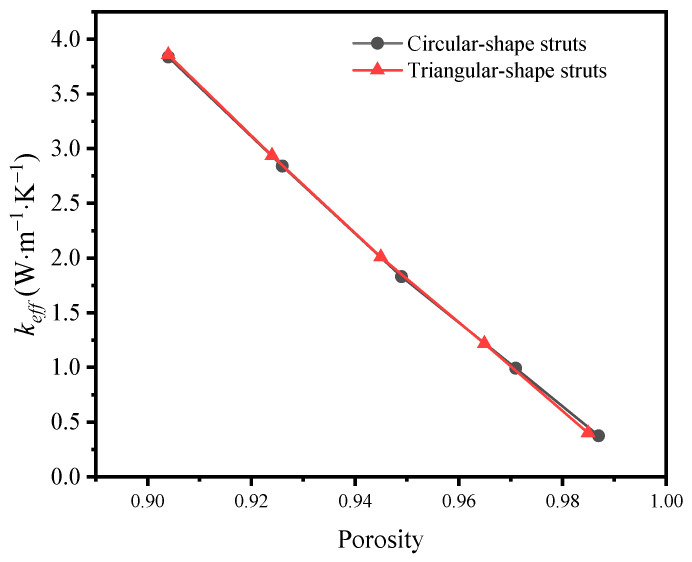
Variation of effective thermal coefficient of circular-shape and triangular-shape struts with different porosities.

Another characteristic of different templates is the various porosities. When the mass fraction of graphene remains, the porosity is changed via enlarging or reducing the diameter of struts. Figure 6 depicts the influence of porosity on the effective thermal coefficient of the GF composites with circular-shape and triangular-shape struts individually. In the big picture, the effective thermal coefficient drops with rising porosity. These results agree well with those of other researchers. Chan et al. [40] found that the effective thermal conductivity of graphene-coated metal foams was decreased from 7 W·m^−1^·K^−1^ to 4 W·m^−1^·K^−1^ when the porosity was increased from 0.7% to 0.98%. AL-Saleem et al. [41] also found that the thermal conductivity of nickel foam coated with 200 nm graphene was decreased from 4.12 W·m^−1^·K^−1^ to 3.58 W·m^−1^·K^−1^ as the pore size decreased from 725.7 μm to 195 μm. That was because the larger the porosity, the smaller the volume of the solid skeleton involved in heat conduction, resulting in a smaller effective thermal conductivity. In detail, the cross-section shape has little impact on the thermal coefficient at a similar porosity, although it disturbs the temperature distribution. Hence, the porosity of the growth template is more significant than the cross-section shape to the thermal properties of GF composite.

#### 3.1.2. The Impact of the Growth Template on Thermal Properties

When the deposition rate remains the same, the deposition thickness of graphene depends on the deposition time, which simultaneously increases the mass fraction of graphene. Is it good or not to take a longer time to deposit graphene? Pettes et al. [28] provided the total thermal conductivity of GF at different deposition thicknesses of graphene. The experimental results proved that the heat transfer was enhanced with the increase of graphene layers when the total number of layers was small, but the heat transfer was worse with the graphene being thickened continuously. Liu et al. [39] explained the enhancement of thermal conductivity in the initial phase was owed to the decrease of graphene interlayer spacing according to the experiment and the calculation based on Bragg’s law. As the distance between the interlayers is equivalent to the thickness of monolayer graphene and is reduced, the thermal contact resistance shrinks with the overlaying graphene. With the accumulation of thermal contact resistance, the total thermal conductivity declines after climbing to a peak. Therefore, the curve of effective thermal coefficient tends upward firstly and then downward with the increase of the graphene mass fraction.

**Figure 7:**
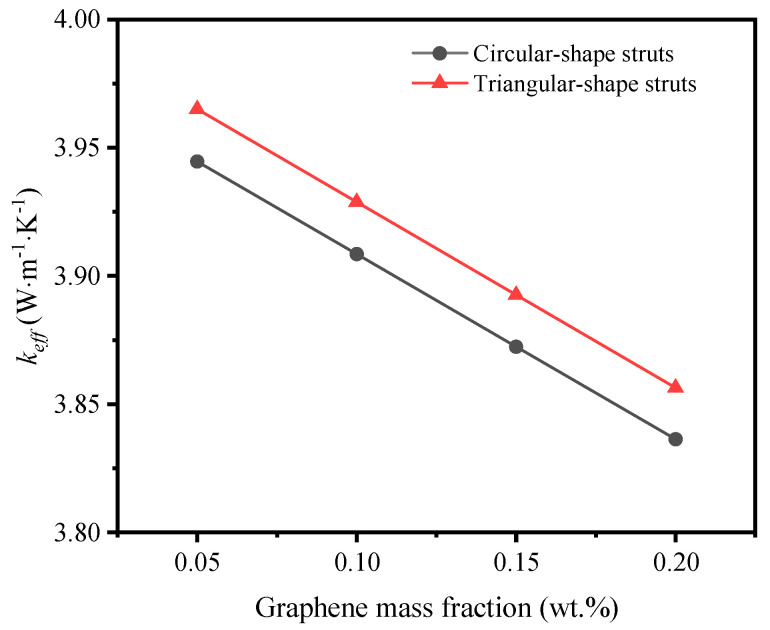
Variation of effective thermal coefficient with graphene mass fraction.

As the preparation of a monolayer or several layers of graphene deposition is difficult, and it is hard to achieve uniform deposition with a low number of layers, here we adopted the thin shell element method to explore the thermal properties of graphene with a layer-by-layer deposited feature and continuous scale wall thickness. Figure 7 shows the effective thermal coefficient decreases with the increase of graphene mass fraction, no matter what the shape of the cross section is. The slope of both curves stays similar. However, the value of the effective thermal coefficient of triangular-shape struts is higher than that of circular-shape struts. That is because the total surface area of circular-shape struts is smaller than that of triangular-shape struts when the solid volume fraction is the same, which makes the graphene wall thicker on the circular-shape struts. In this view, the deposition thickness of graphene dramatically influences the thermal properties of the GF composite, and magnifies the effect of the cross-section shape of struts on the heat transfer. It guides the control of deposition time in the fabrication experiment.

### 3.2. The Thermal Properties and the Influence Factors at a High Temperature

As the radiative thermal energy is proportional to the fourth power of temperature according to the Stefan–Boltzmann law, the contribution of thermal radiation cannot be ignored at a high temperature. In open-cell foam materials, there are many solid surfaces participating in radiation heat transfer, and the shielding relationship between the skeletons is complex, resulting in a significant impact of thermal radiation on heat transfer. The coupled conductive–radiative heat transfer of foam composite with 0.2 wt.% graphene was therefore investigated at high temperature.

#### 3.2.1. The Impact of Mean Temperature on Thermal Properties

**Figure 8:**
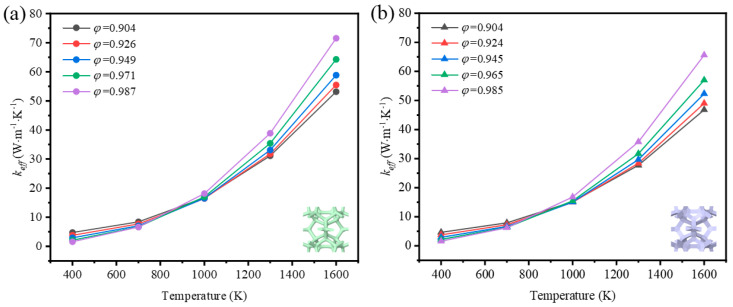
Effective thermal coefficient of GF composite depending on the mean temperature: (**a**) circular-shape struts, (**b**) triangular-shape struts.

The effective thermal coefficient of GF composite depending on the mean temperature is shown in Figure 8. The mean temperature was defined as *T_ave_* = (*T_H_* + *T_L_*)/2, where TH and TL are the temperature of the top and the bottom surfaces. The emissivity of graphene was 0.9 [28]. For the GF composite with different porosities, the effective thermal coefficient grew nonlinearly with the mean temperature rising, and the nonlinearity was much more remarkable when the mean temperature was higher. In the range of room temperature, the heat transfer of low-porosity foams is better than that of high-porosity foams, as the thermal conductivity of solid rules. However, the heat transfer develops in a different trend so that the effective thermal coefficient of high-porosity foam is much higher than that of low-porosity foam with the temperature rising. This demonstrates that thermal radiation plays a significant role at a high temperature.

**Figure 9:**
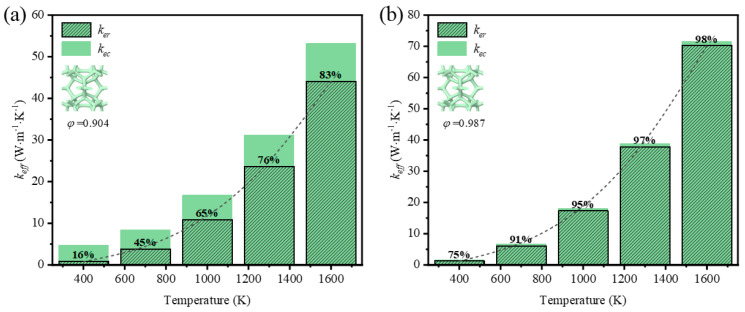
Variation of effective thermal coefficient and thermal radiation contribution with temperature in GF composite with circular-shape struts at different porosities (*φ*): (**a**) *φ* = 0.904; (**b**) *φ* = 0.987.

**Figure 10:**
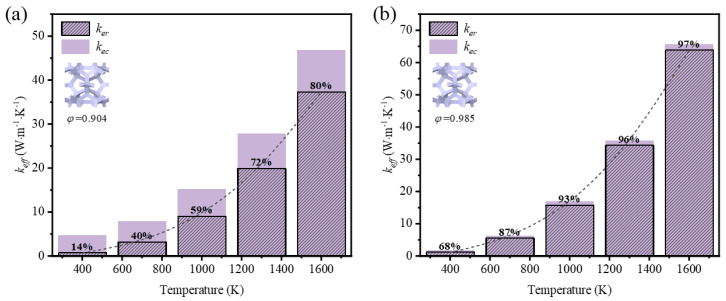
Variation of effective thermal coefficient and thermal radiation contribution with temperature in GF composite with triangular-shape struts at (**a**) *φ* = 0.904; (**b**) *φ* = 0.985.

To reveal the effect of thermal radiation on the total heat transfer, the contribution of both heat transfer methods is illustrated in Figure 9 and Figure 10. It is seen that the thermal radiation accounted for 16–83% with the temperature rising at low porosity (*φ* = 0.904) in the foam with circular-shape struts, and from 14–80% in the foam with triangular-shape struts. The big leap in the proportion discloses the essential change in the way heat was transferred and the importance of thermal radiation. Relatively, thermal radiation occupied a large scale at the initial high porosity (*φ* = 0.987 and *φ* = 0.985) in the foam with different-shape struts, and the effective thermal coefficient surpassed that of the foam with low porosity under the influence of radiation. Thus, the mean temperature can dramatically change the thermal properties of GF composite and the function of applications. This regulation showed more prominently in the foam with higher porosities, where the ratios of effective thermal coefficient at a high temperature to that at a room temperature were 44.5 and 39.7, respectively, for the circular-shape struts and the triangular-shape struts. In addition, the ratio of radiative thermal conductivity to effective thermal conductivity of the model with a porosity of 0.987 at an average temperature of 1600 K was 98%. Shahrzadi et al. [42] also found that the radiation contribution of a body-centered cubic lattice model was affected by the porosity. When the average unit cell temperature was 1800 K, the porosity and the ratio of radiative thermal conductivity to effective thermal conductivity were 0.99 and 95%, respectively. It can be concluded that the pore structure of graphene foam composite is more strongly affected by the radiative thermal conductivity than that of the BCC lattice structure.

#### 3.2.2. The Impact of Porosity on Thermal Properties

**Figure 11:**
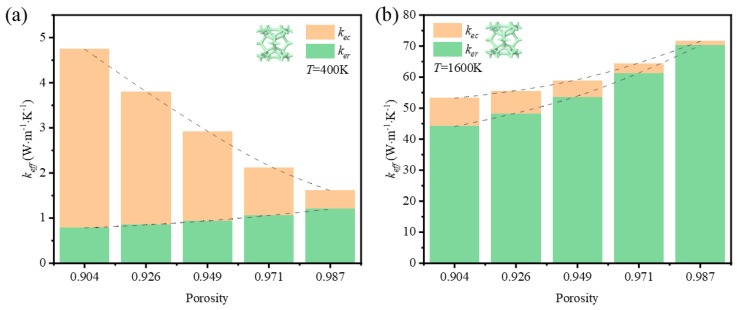
Variation of effective thermal coefficient and contribution of thermal radiation with porosity of foam with circular-shape struts at (**a**) *T* = 400 K; (**b**) *T* = 1600 K.

**Figure 12:**
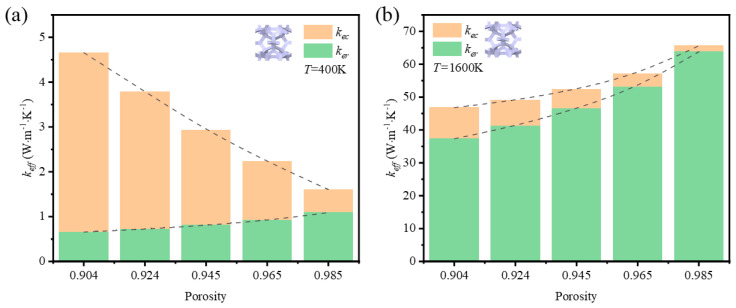
Variation of effective thermal coefficient and contribution of thermal radiation with porosity of foam with triangular-shape struts at (**a**) *T* = 400 K; (**b**) *T* = 1600 K.

In addition to the research on the impact of porosity on the thermal properties at room temperature, the impact of porosity at 400 K and at 1600 K is compared in Figure 11 and Figure 12.

Regardless of the influence of the strut shape and the temperature, the contribution of thermal radiation simultaneously increased with the increase of porosity. This agrees with the research on thermal radiation contribution to the effective thermal coefficient of metal foams [43]. At 400 K, the drop of thermal conductivity of the solid skeleton was much faster than the increase of thermal radiation with rising porosities. The whole trend of effective thermal coefficient was down, which demonstrates that thermal conduction dominates the thermal properties of GF composite at room temperature. At 1600 K, the contribution of thermal radiation always occupied a leading position from low to high porosity, which resulted in an increased rate of both thermal radiation and thermal conduction that was not dramatic. However, the fact that the thermal radiation was enhanced in the foam with high porosity demonstrates the surface area is not the main factor to determine the thermal radiation, because the diminishment of porosity was realized via reducing the effective diameter of struts. The enhancement might come from the enlargement of visible surface area, which affects the view factor directly. In sum, the porosity is important to the heat transfer, especially to the thermal radiation at a high temperature.

### 3.3. The Impact of Cross-Section Shape of Struts on Thermal Properties

**Figure 13:**
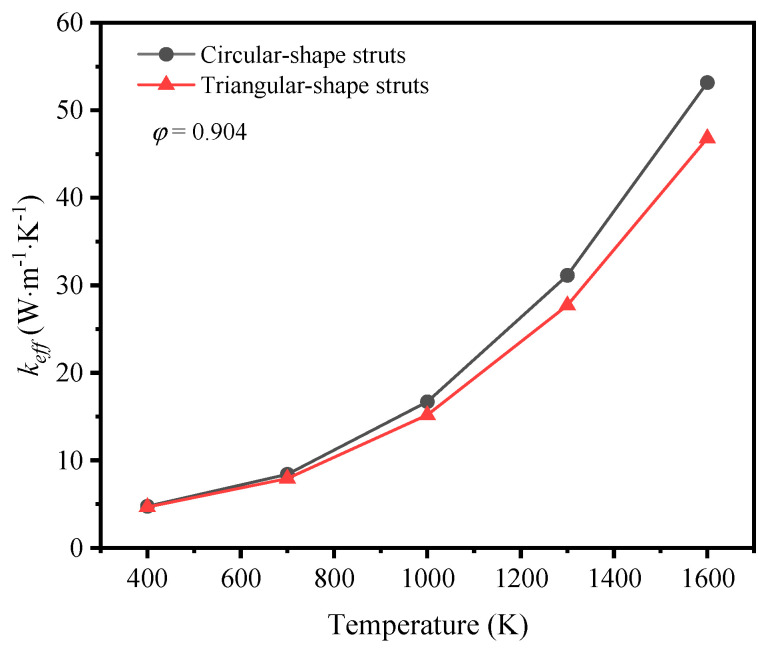
Variation of effective thermal coefficient with temperature.

The variation of the effective thermal coefficient of GF composite with different cross-section shaped struts with the mean temperature is shown in Figure 13 at a porosity of 0.904. Besides the same non-linear growth trend, the gap of effective thermal coefficient is enlarged with the temperature rising. The effective thermal conductivity of the GF with circular-shape struts increases more significantly with the increase of temperature in comparison with that of GF with triangular-shape struts.

**Figure 14:**
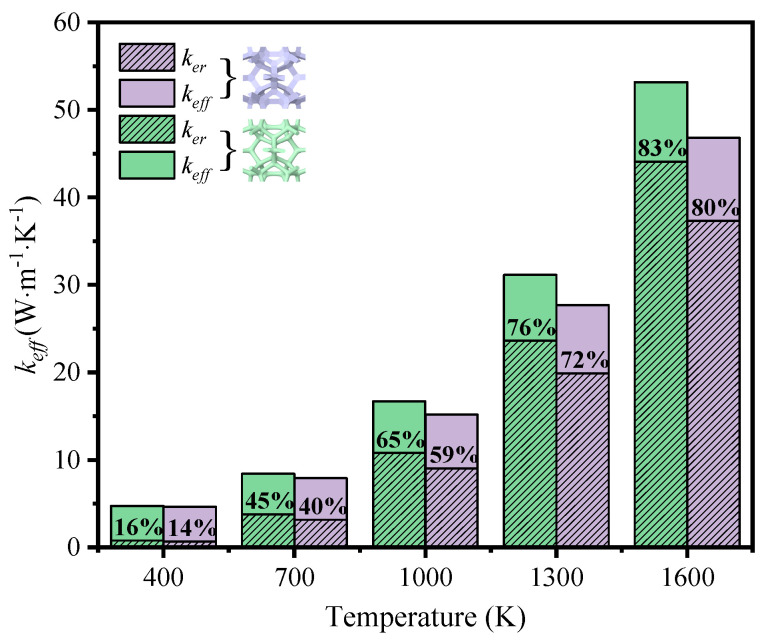
Contribution of thermal radiation to effective thermal coefficient of the foam with different strut shapes at variable temperatures.

Figure 14 explains the difference of the development depending on temperature. It was found that the proportion of thermal radiation of the foam with circular-shape struts processed faster than that of the foam with triangular-shape struts, which was the reason for the widening gap of effective thermal coefficient between the foams with different strut shapes, despite the similarity in value at low temperature.

### 3.4. The Impact of Cross-Section Shape of Struts on Thermal Properties

In the coupled conductive–radiative heat transfer, the emissivity affects the emission and absorption of the skeleton surface according to Kirchhoff’s law. The emissivity is determined by the intrinsic radiation property and the morphology and structure of the surface mostly, which are directly influenced by the choice of growth template, deposition conditions, and etching solutions during the preparation of GF by the CVD method. In this research, the impact of emissivity varying from 0.6 to 1.0 was investigated at the mean temperature of 1600 K on the effective thermal coefficient of GF composites with circular-shape and triangular-shape struts at low and high porosities. 

**Figure 15:**
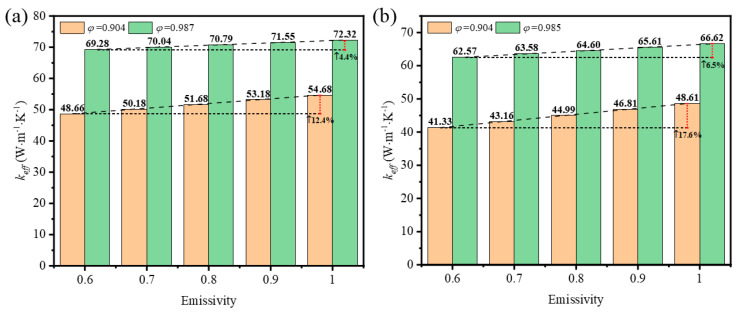
Variation of effective thermal coefficient with emissivity at different porosities of the foam with (**a**) circular-shape struts; (**b**) triangular-shape struts.

Figure 15a shows the effective thermal coefficient increased by 12.4% and 4.4% with the increase of emissivity of the foam with circular-shape struts at porosity values of 0.904 and 0.987. The increase was 17.6% and 6.5%, respectively, for foam with triangular-shape struts at porosity values of 0.904 and 0.985, as shown in Figure 15b. This demonstrates that the increase of the effective thermal coefficient at low porosity was larger than that at high porosity; the increase in the foam with triangular-shape struts was also larger than that in the foam with circular-shape struts. The explanation for the difference of increase is that the surface area of the foam with low porosity and triangular-shape struts was greater than that of the foam with high porosity and circular-shape struts, which enhanced the thermal radiation overall.

### 3.5. The Impact of Temperature Difference on Thermal Properties

**Figure 16:**
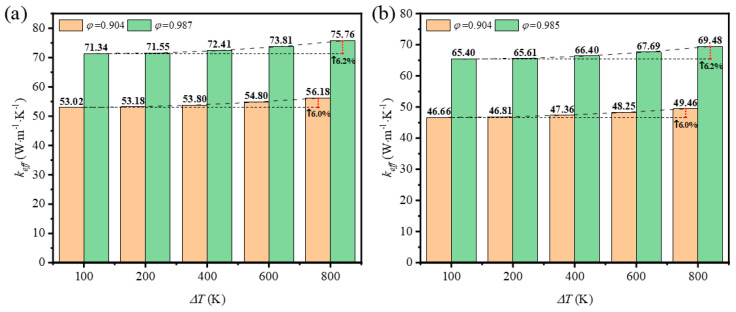
Variation of effective thermal coefficient with temperature imposed on both surfaces at different porosities of the foam with (**a**) circular-shape struts; (**b**) triangular-shape struts.

In previous studies on the effective thermal conductivity of GF composite, most of the focus was on the effect of average temperature on the thermal conductivity. Many experiments used a fixed temperature gradient applied on both sides of the sample. Few studies focused on the effect of the temperature difference on the two sides of the sample on the effective thermal conductivity. As the mean temperature is the geometrical mean of temperature on the top and bottom surfaces, here we set the temperature difference as from 100 K to 800 K when the mean temperature remained at 1600 K. The results in Figure 16 show that the effective thermal coefficient is influenced by the temperature difference. Compared with Figure 16a,b, it is seen that the strut shape has no obvious effect on the results and the impact of porosity here is also tiny, but the temperature difference really determines the variation of effective thermal coefficient to some extent. Although the enhancement of heat transfer is limited, the impact should be admitted.

### 3.6. Curve Fitting

According to the calculated data, the mathematical expressions of effective thermal conductivity, porosity, and temperature were further fitted. Kovács et al. [44] proposed a second-order polynomial of the effective thermal conductivity as a function of the porosity over the entire range of 0 ≤ *φ* ≤ 1:

(16) $$k_{\mathrm{eff}}=a_{0}\varphi^{2}+a_{1}\varphi +a_{2}$$

where *φ* is the porosity.

Based on this polynomial, the mathematical expression of the effective thermal conductivity and porosity without considering radiation effect is fitted as:

(17) $$k_{\mathrm{eff}}=75.192\varphi^{2}-184.05\varphi +108.79$$

**Figure 17:**
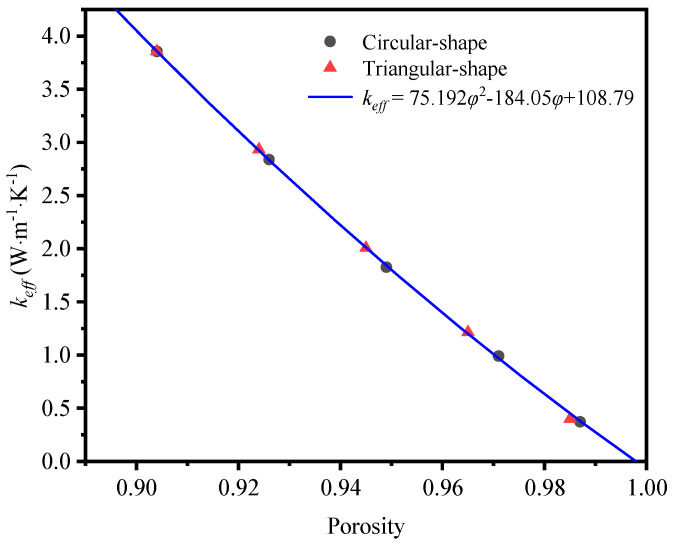
Comparison between effective thermal conductivity in simulated results and correlation in Equation (17).

Figure 17 shows the comparison of effective thermal conductivity between simulated results and correlation in Equation (17) under the condition of neglecting thermal radiation. It can be observed that the fitted mathematical equation is in good agreement with the simulation data.

When thermal radiation is taken into consideration at high temperature, the effective thermal conductivity of GF composite highly depends on porosity and temperature. Therefore, the effective thermal conductivity can be expressed as the function of composition of porosity and temperature as follows:

(18) $$k_{\mathrm{eff}}=\left(\varphi ,T\right)$$

According to the effective thermal conductivity of the GF composite obtained at different porosities and temperatures, the effective thermal conductivity as the function of porosity and temperature can be expressed by:

(19) $$k_{\mathrm{eff}}=(0.0001857{-0.00013595\varphi )T}^{2}-(0.1964\varphi -0.1506)T\;+\left(571.93\varphi^{2}-1070\varphi +510.8\right)$$

**Figure 18:**
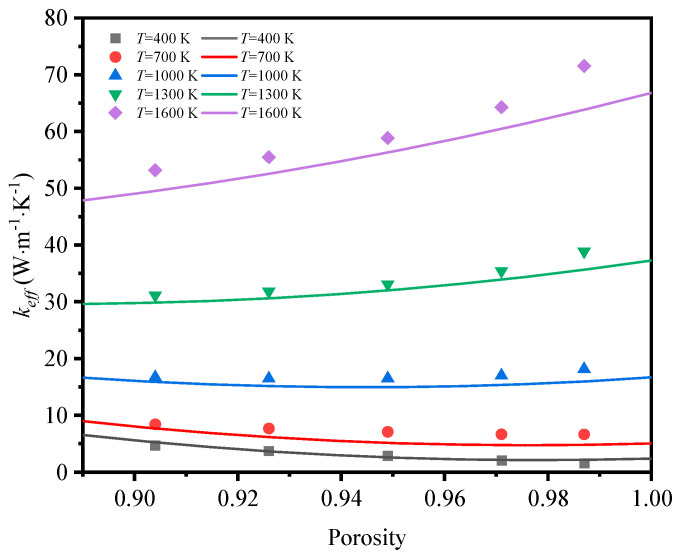
Comparison between effective thermal conductivity in simulated results and correlation in Equation (19).

Figure 18 shows the comparison between the correlation in Equation (19) and the simulated results when the thermal radiation is taken into consideration. It can be observed that the fitting mathematical equation is in high agreement with the simulated results at different temperatures and porosities.

## 4. Conclusions

In this study, the finite element method was applied to numerically investigate the thermal conductivity and radiation processes of GF composite prepared by the CVD method under different experimental preparation parameters. The influences of the skeleton cross-section shape, porosity, temperature, and other parameters on the effective thermal conductivity were studied, as well as the contribution of thermal radiation to the effective thermal conductivity under different parameters. The main findings can be summarized as follows:

(1) The effective thermal conductivity of GF composite can be enhanced with the decrease of porosity, which can be attributed to the higher solid volume fraction at lower porosity. In addition, the effective thermal conductivity of GF composite is decreased with the increase of deposition time. When the volume fraction of graphene increased from 0.05% to 0.2%, the effective thermal conductivity of the GF composite decreased from 3.96 W·m^−1^·K^−1^ to 3.86 W·m^−1^·K^−1^.
(2) Under the condition of temperature higher than 400 K, the thermal radiation effect cannot be neglected. At a given porosity of 0.904, when the average temperature increases from 400 K to 1600 K, the effective thermal conductivity of GF composite with circular sections and triangle sections increased by 1021.94% and 906.45%, respectively. In addition, GF composite with higher porosity was more easily affected by temperature compared to that with lower porosity. For GF composite with circular cross-sections at 1600 K, the contribution of thermal radiation to effective thermal conductivity increased from 83% to 98% when the porosity increased from 0.904 to 0.987.
(3) The thermal properties of GF composite with lower porosity and larger pore surface area are more sensitive to changes of surface emissivity. When the surface emissivity of GF composite increased from 0.6 to 1, the effective thermal conductivity of GF composite with circular cross-section struts increased by 12.4% and 4.4% at porosity values of 0.904 and 0.987, respectively. The effective thermal conductivity of GF composite with triangular cross-section struts increased by 17.6% and 6.5% at porosity values of 0.904 and 0.985, respectively.

Therefore, the effective thermal conductivity of GF composite at temperature lower than 400 K can be enhanced by reducing the growth template porosity, adjusting the deposition time, and controlling the number of graphene layers. At temperature higher than 400 K, the effective thermal conductivity of GF composite can be improved by increasing the temperature and growth template porosity, reducing the surface area and increasing the surface emissivity. Compressibility is another critical characteristic of graphene foam composite in practical applications. Therefore, the influences of compressibility on the thermal conductivity of graphene foam composite will be considered in our future research work.

## Figures and Tables

**Table 1:** Thermophysical properties of materials used in the simulation.

Material	Thermal Conductivity (W·m^−1^·K^−1^)	Specific Heat (J·kg^−1^·K^−1^)	Density (kg·m^−3^)
Air	0.026	1006.43	1.225
Graphene	500	709	2250
Nickel	106	460.6	8900
Epoxy resin	0.2617	1110	1450

## Data Availability

Data are contained within the article.
